# An obesity educational intervention for medical students addressing weight bias and communication skills using standardized patients

**DOI:** 10.1186/1472-6920-14-53

**Published:** 2014-03-18

**Authors:** Robert F Kushner, Dinah M Zeiss, Joseph M Feinglass, Marsha Yelen

**Affiliations:** 1 Division of Endocrinology, Metabolism, and Molecular Medicine, Northwestern University Feinberg School of Medicine, 750 North Lake Shore Drive, Rubloff 9-976, Chicago, IL 60611, USA; 2 Division of General Internal Medicine, Northwestern University Feinberg School of Medicine, 750 North Lake Shore Drive, Rubloff 10th Floor, Chicago, IL 60611, USA; 3 Office of Medical Education, Northwestern University Feinberg School of Medicine, 240 East Huron Street, Chicago, IL 60611, USA

**Keywords:** Obesity, Standardized patient, Stigma, Communication, Empathy, Medical students

## Abstract

**Background:**

In order to manage the increasing worldwide problem of obesity, medical students will need to acquire the knowledge and skills necessary to assess and counsel patients with obesity. Few educational intervention studies have been conducted with medical students addressing stigma and communication skills with patients who are overweight or obese. The purpose of this study was to evaluate changes in students' attitudes and beliefs about obesity, and their confidence in communication skills after a structured educational intervention that included a clinical encounter with an overweight standardized patient (SP).

**Methods:**

First year medical students (n = 127, 47% female) enrolled in a communications unit were instructed to discuss the SPs' overweight status and probe about their perceptions of being overweight during an 8 minute encounter. Prior to the session, students were asked to read two articles on communication and stigma as background information. Reflections on the readings and their performance with the SP were conducted prior to and after the encounter when students met in small groups. A newly constructed 16 item questionnaire was completed before, immediately after and one year after the session. Scale analysis was performed based on *a priori* classification of item intent.

**Results:**

Three scales emerged from the questionnaire: negative obesity stereotyping (7 items), empathy (3 items), and counseling confidence (3 items). There were small but significant immediate post-intervention improvements in stereotyping (p = .002) and empathy (p < .0001) and a very large mean improvement in confidence (p < .0001). Significant improvement between baseline and immediate follow-up responses were maintained for empathy and counseling at one year after the encounter but stereotyping reverted to the baseline mean. Percent of students with improved scale scores immediately and at one year follow up were as follows: stereotyping 53.1% and 57.8%; empathy 48.4% and 47.7%; and confidence 86.7% and 85.9%.

**Conclusions:**

A structured encounter with an overweight SP was associated with a significant short-term decrease in negative stereotyping, and longer-term increase in empathy and raised confidence among first year medical students toward persons who are obese. The encounter was most effective for increasing confidence in counseling skills.

## Background

A major challenge facing medical educators today is to adequately train current and future physicians in the prevention and treatment of chronic illnesses. Underlying this health threat is the alarming increase in the number of adults and children who are overweight and obese. In 2007 the Association of American Medical Colleges (AAMC) published a Contemporary Issues in Medicine Report VIII report titled *The Prevention and Treatment of Overweight and Obesity*[1]. The report concluded by stating “Medical education must assume that future physicians will be better prepared to provide respectful, effective care of overweight and obese patients and to appropriately participate in overweight/obesity prevention efforts. Education on assessing, preventing and treating overweight and obesity should be included in basic science, clinical experiences, and population health sciences.” [1]. The report directly addresses the need to develop a competent and knowledgeable physician workforce in the twenty-first century that can provide care to the 66% of the U.S. adults and 33% of U.S. children and adolescents who are either overweight or obese [2,3]. This ‘call to action’ was also the topic of a recent editorial on the need to educate physicians-in-training to manage obesity [4].

Yet despite the enormity of the health problem, few educational intervention studies have been conducted in U.S. medical schools addressing knowledge, attitudes, or skills regarding obesity. In a recent systematic review, Vitolins et al. [5] identified only five publications from a PubMed search conducted between 1966 to 2010 that included predefined obesity intervention and evaluation elements. They concluded that there were very few published studies that reported the effectiveness of medical school obesity educational programs. In another systematic review to investigate how effective educational interventions are in preparing medical students to facilitate lifestyle changes with obese patients, Chisholm et al. [6] conducted an extensive database search for articles published between 1990 and 2010 and identified 12 educational studies that met their predefined eligibility criteria. Only five studies were exclusively related to educational interventions on obesity itself. Due to lack of robust evaluations, transparency of methods and presence of potential bias, the authors could not determine the efficacy of the interventions. They concluded that more work was needed to develop and identify evidence-based educational interventions about obesity-related lifestyle change.

Two topics in obesity education that particularly merit increased attention are stigma and communication skills. In a recent study by Miller et al., 354 third year medical students completed the Weight Implicit Association Test (IAT) and a semantic differential item assessing their explicit preferences for fat or thin individuals [7]. Overall, approximately 40% of students had a moderate or strong implicit anti-fat bias, yet few were aware of it. Patients with obesity are also a common target of derogatory humor by students, attendings and residents [8]. In addition, survey data shows that physicians harbor negative attitudes toward the obese despite an appreciation for the complexity of the condition [9-12]. These negative attitudes may influence the quality of healthcare provided to patients with obesity and willingness to engage in weight management. Other data repeatedly show that discussions between physicians and patients surrounding body weight and obesity only occur in a minority of encounters [13-17]. It can be hypothesized that tackling these attitudes and skills regarding obesity during undergraduate education may increase the preparedness of physicians to provide more frequent and empathetic obesity care. During the first year of medical school, standardized patients (SPs) provide an important opportunity for learning communication skills in a safe and controlled environment along with formative feedback [18]. Although SPs have been used to facilitate development and assessment of behavior change skills among medical students and physicians [19-21], their use in addressing stigma has not been explored. The purpose of this study was to evaluate changes in students' attitudes and beliefs about obesity, and their confidence in communication skills after undergoing an educational intervention that included a structured clinical encounter with an overweight SP.

## Methods

The study was conducted at Northwestern University Feinberg School of Medicine (NUFSM) in Chicago, IL and approved by the Northwestern University Institutional Review Board. In the fall semester Communication Skills unit for first year medical students, SPs are utilized to help teach fundamental communication skills such as setting the stage, eliciting information, giving information and counseling for health promotion [22]. For the class of 2015, a session was deliberately developed that directly addressed communication skills with an overweight or obese patient. Twelve SPs already selected and trained to work with students for the Communication Skills unit and who identified themselves as either being overweight (n = 8) or had a family member who struggled with obesity (n = 4) were selected for this particular session. All SPs participating in the Communication Skills unit were experienced SPs and received extensive training prior to the start of the unit which included; understanding the weekly student learning objectives, giving verbal feedback on students communication skills, facilitating group discussion regarding communication skills, patient affect and strategies in using case facts to role play the patient scenario. The SP training was conducted by one of the Clinical Education Center SP trainers who have extensive experience in medical education.

Six short, loosely structured patient scenarios were created for role playing to provide a broad range of realistic physician-patient encounters (Table 1). SPs were expected to elaborate with responses that seemed natural to them since they already had knowledge and/or experience with the subject. For training, SPs were sent the scenarios ahead of time and asked which one(s) they would be comfortable role playing. From the case selections made, SPs were trained by the SP trainer in small groups for one hour at which time they each role played the scenarios assigned to them. During the training, the SP trainer coached the SPs (as needed) to respond in a natural way with a realistic affect. Prior to the encounter, students were asked to read two short articles that were posted on the electronic blackboard that focused on communication issues about weight [23] and obesity stigma [24]. After a brief (15 minute) review of the articles with the faculty preceptor, students were instructed to discuss the SPs' perception of their weight, take a weight history and probe for how their weight has affected them socially and physically. Each student (in groups of 3 or 4) conducted an 8 minute encounter with the SP followed by 8 minutes debriefing in which the students received formative feedback on their communication skills. The feedback was provided by the SP and other students regarding their performance. The student who performed the interview first discussed what went well during the SP encounter. Afterwards, other students in the group offered their comments, followed by the SP. The same format was used again regarding communication skills that could have been done differently or improved by the interviewer. SPs comments were focused on their observations of verbal and non-verbal communication and how it made him/her feel from a patient’s point of view. SPs rotated from room to room offering each student the opportunity to interview a different SP role playing a different scenario, followed by the feedback process. Each student group was able to observe up to four of the six scenarios. After the SP encounter activity, students met once again with the faculty preceptor for an additional 30 minutes of facilitated reflection and discussion of the SP interaction. At the start of the unit, small group faculty preceptors met with the unit director for an orientation session that included review of learning goals and objectives, curricular content, and the assessment and evaluation of student performance based on participation, knowledge, skills and attitudes. Although empathy building was an overarching goal of the unit, the preceptors did not receive special training in weight bias.

**Table 1:** Patient scenarios used by the standardized patients

**Number**	**Scenario**
1	Patient has never thought about losing weight and doesn’t consider herself having a weight problem
2	Patient knows she has a weight problem, has tried losing weight on multiple occasions but finds it hard to manage long-term
3	Patient had a bad experience in the past with doctors that made her feel ashamed and humiliated
4	Patient is hesitant to talk about her weight since it makes her feel bad. She does not like her body shape and size
5	Patient did not know her weight was a medical problem and wants to learn how she can take control
6	Patient comes from an obese family and assumes that it is all genetic and nothing will work

To assess student attitudes, beliefs and confidence regarding interaction with an obese person, existing relevant surveys from the medical literature were reviewed [9,25-27]. In order to capture the specific outcome variables of interest for this study and to limit the length of the questionnaire, selected items from these surveys were chosen to create a new 16-item, 5-point Likert scale questionnaire ranging from strongly agree (5) to strongly disagree (1). One week before the encounter and reading the two articles, students completed the baseline pre-questionnaire. The post-questionnaire was completed at two time points: shortly after concluding the SP encounter (immediate) and one year following the encounter (long-term). In addition, two items were included on the one year follow up questionnaire to explore students’ long-term perceptions of the encounter. Using the same 5-point Likert scale, students responded to two statements: “The session had a long-lasting influence on the way I think about obesity or an obese patient” and “The session had a long-lasting effect on my comfort level to talk with obese patients.”

Scales were derived empirically from the baseline questionnaire based on grouping and summing (1 = strongly disagree to 5 = strongly agree) items by their focus on student’s potential stereotypes, ability to overcome stigma, and confidence in their ability to counsel. After grouping items accordingly, scale properties were tested using Cronbach’s alpha to determine reliability. Three reliable scales emerged reflecting: seven items that elicited ratings of negative obesity *stereotypes* (alpha = 0.78) where a higher score indicated greater stereotyping; three items rating *empathy* for obese patients (alpha = 0.63); and three items positively rating students’ *confidence* on their ability to clinically interact with obese patients (alpha = 0.60). Three additional items did not contribute to any of the scales. The scales were not significantly correlated with each other. All three scales were approximately normally distributed at baseline. Mean differences in each scale between each test instance were analyzed using a paired two-tailed t-test.

## Results

First year medical students (n = 127) from NUFSM who were enrolled in the Communication Skills unit and completed all three questionnaires were included in the study. The completion rate was 81% for total class enrollment of 157 students. Students were 24 ± 2.8 years old, 47% female; 50% white, 22% Asian, 11% Hispanic and 4% Black. Self-report data on student’s height, weight and computed body mass index (BMI) was also obtained; 15.3% of the sample had a calculated BMI > 25 kg/m^2^. The newly created 16-item questionnaire is shown in Table 2. For the scaled analysis we summed the questions into the 3 scale scores: Stereotyping (items 1, 2, 5, 6, 7, 8 and 9), Empathy for obese patients (items 10, 11 and 12), and Confidence in clinical interaction with obese patients (items 14, 15 and 16). Mean scale scores are presented in Table 3 weighted for the number of items to reflect a single Likert item scale. Mean differences on the three scales between the baseline questionnaire compared to the immediate and long-term post-questionnaires are also presented. Significant improvement between baseline and the immediate follow-up survey was observed in all three scales, ranging from small improvement in stereotyping and empathy, and a much larger mean improvement in confidence. Over fifty-three percent of students indicated less obesity stereotyping (versus 32.8% who indicated greater stereotyping) based on a declining score, 48.4% indicated more empathy for obese patients (versus 23.4% who indicated less), and 86.7% showed more confidence in clinical interaction with obese patients (versus only 7.8% whose confidence declined). At one year, negative obesity stereotyping had regressed to baseline levels and the modest decrease in stereotyping at the immediate follow-up survey had disappeared. However, gains were maintained for the mean empathy and counseling scale scores which remained statistically significant from baseline. For the follow up item, “The session had a long-lasting influence on the way I think about obesity or an obese patient”, 35% of students indicated “strongly agree or agree” while 33% indicated “strongly disagree/disagree”. For the second follow up item, “The session had a long-lasting effect on my comfort level to talk with obese patients” 40% of students indicated “strongly agree or agree” while 25% indicated “strongly disagree or disagree” (p < 0.05 by chi-square test).

**Table 2:** Sixteen item questionnaire used to assess student attitudes, beliefs and counseling confidence with an overweight or obese standardized patient

**Scale**	**Items**
	1. Obese individuals have lower will-power than non-obese people
	2. Individuals are obese due to making poor personal choices
	3. Life events and our environment make weight loss difficult
Negative Obesity Stereotypes	4. Obesity is complex, due to genetics, biology and behavior
	5. Obese individuals are lazier than non-obese people
	6. Obese people are more emotional than non-obese people
	7. Obese individuals don't make good decisions
	8. Obese individuals have themselves to blame
	9. Obese individuals are generally not assertive enough
	10. Obese people feel stigmatized in our society
Empathy for Obese Patients	11. Obese people feel stigmatized by the medical profession
	12. Very few obese are ashamed of their weight
	13. I am uncomfortable being around obese people
Confidence in Clinical Interaction with Obese Patients	14. I feel comfortable talking to people about their weight
	15. I know what meaningful questions to ask to take a body weight history
	16. I know what meaningful questions to ask to help obese people manage their weight

Students indicated response using a 5-point Likert scale ranging from strongly agree (5) to strongly disagree (1).

Items 3, 4 and 13 did not improve scale reliability and were therefore not included in the scale analysis.

Item 12 was reversed coded.

**Table 3:** Student attitudes about negative obesity stereotypes, empathy for obese patients and counseling confidence after interview with an overweight or obese standardized patient: average baseline and follow-up scores

**Scale**	**Mean (SD) baseline scale score**	**Mean (SD) immediate post-test scale score**	**Percent of students with improved score from baseline to immediate post-test**	**P value of paired change between baseline and immediate post-test**	**Mean (SD) long-term follow-up scale score**	**Percent of students with improved score from baseline to long-term follow-up**	**P value of paired change between baseline and long-term follow-up**
Negative Obesity Stereotypes	2.31 (0.55)	2.18 (0.57)	53.1%	0.002	2.29 (0.62)	57.8%	0.87
Empathy for Obese Patients	4.02 (0.54)	4.21 (0.57)	48.4%	<0.0001	4.15 (0.47)	47.7%	0.001
Confidence in Clinical Interaction with Obese Patients	2.41 (0.67)	3.61 (0.67)	86.7%	<0.0001	3.39 (0.66)	85.9%	<0.0001

Student responses on scale scores averaged to a 5-point Likert scale ranging from strongly agree (5) to strongly disagree (1).

## Discussion and conlusions

In this study we found that an educational initiative that included an overweight or obese SP along with targeted readings and facilitated discussion specifically designed to address stereotyping, empathy and communication skills during the first year of undergraduate training can have an immediate beneficial impact on all three domains. One of the strengths of this study was the re-evaluation of students in the fall semester of their second year, one year following the Communication Skills unit SP encounter. The re-evaluation allowed us to assess the durability or deterioration of any change that occurred over the year. One-year post-questionnaire scale data showed that students’ empathy, confidence and comfort interacting with an overweight or obese patient remained intact. The percentages of students who showed improved scaled empathy scores compared to baseline were 48.4% (p < 0.001) and 47.7% (p < 0.01) immediately and long-term, respectively. Immediate and one year improvement in counseling was more robust. The corresponding changes in scaled confidence scores were 86.7% (p < 0.001) and 85.9% (p < 0.001). This is encouraging since the perseverance of change scores occurred without introduction of other obesity focused learning activity during the year. The obesity focused session included three related interventions that addressed student confidence; selection of a clinically relevant obesity communication article, guided reflection and discussion in the small group, and the actual SP encounter. Thus, the relative influence of each learning approach could not be determined.

In contrast to empathy and counseling, scaled stereotyping mean scores showed a regression back to baseline over the year. Although 53.1% of students improved immediately after the SP encounter and 57.8% improved at long-term follow up, change was no longer statistically significant (p < 0.87). The reason for the lack of significance is because the mean of improved scores declined -2.5 total points while the mean of the same or increased scores increased 3.3 points. Thus while more students continued to show improved (declining) stereotype attitudes the overall mean scale score reverted back to baseline. Although we did not probe for reasons that underlie the changed stereotyping scores during the follow-up, it is possible that some students were beginning to reflect attitudes and perceptions they encountered during their first year in medical school which included clinical exposures in health screens, volunteer time in health clinics, and with their clinical preceptors. Bias and obesity stigma has been well described among physicians [11,28] and is it possible that our clinical instructors hold similar views. Alternatively, the SP encounter was seen as an observable educational activity and did not reflect other obese patients they routinely saw in their clinical environments. Furthermore, it supports the need for additional educational experiences in the medical curriculum. Recently a new 31 item scale to measure medical student attitudes and beliefs regarding obesity was developed that initially appears to have good validity and reliability [29]. However, it was not available at the time of our study.

Other educational initiatives have been conducted among medical students that address various aspects of the obesity encounter, most notably lifestyle behavior change, treatment strategies, conditions related to obesity, and communication skills [4,5]. In particular, standardized patients are commonly used to develop and assess communication training that includes skills in relationship building, patient assessment and behavior change [18-21,30]. To our knowledge educational initiatives to reduce stigma have not been addressed using a SP encounter. Stigma is a particularly important topic to address early in medical education since the obese patient is a frequent target for offensive comments among medical students. In a focus group study conducted by Wear et al. [8], among 58 third- and fourth-year medical students, morbidly obese patients were singled out as the most common target of derogatory humor by students, attendings and residents. Using videotaped simulating cases, Wigton and McGaghie [31] previously demonstrated that the appearance of obesity alone biased students’ impressions of the patient and the patient’s ability to comply with lifestyle recommendations. In one of the few studies that directly examined the patient’s weight on medical students’ attitudes, beliefs and interpersonal behavior, Persky and Eccleston [32] randomly assigned 76 clinical-level medical students to interact with a digital, virtual female patient that was visibly either obese or non-obese. Measurements obtained after the encounters showed significantly higher levels of negative stereotyping, less anticipated patient adherence, worse perceived health, and more responsibility attributed for potentially weight-related presenting complaints directed toward the obese version of the virtual patient than the non-obese version. Thus, medical students express many of the same negative attitudes toward obese patients as physicians [33].

There has been a paucity of studies that have addressed anti-fat bias among medical students or other preservice health students. Wiese et al. [34] used a brief intervention that included an empathy evoking video, written messages about uncontrollable causes of obesity, and role-playing among first-year medical students. Although they were able to demonstrate changes in beliefs about obesity and less anti-fat stereotyping compared to the control group, no significant differences were found at one-year. O’Brien et al. [35] randomized health promotion/public health students to one of three 5-week tutorial conditions: an obesity curriculum on the controllable reasons for obesity, a prejudice reduction condition presenting evidence on the uncontrollable reasons for obesity, and a neutral curriculum on alcohol use. Measures of implicit and explicit anti-fat prejudice, beliefs about obese people, and dieting, were taken at baseline and post-intervention. The authors found that anti-fat prejudice was modestly reduced by focused education about uncontrollable reasons for obesity but exacerbated by focused education about controllable reasons for obesity. Using a pre-post experimental design, Swift et al. [36] investigated the effects of viewing brief educational films specifically designed to reduce weight stigmatization toward obese patients among dietetic and medical students. At baseline, participants demonstrated weight bias as well as strong beliefs that obesity is under a person’s control. The intervention significantly improved explicit attitudes and beliefs toward obese people, however the intervention did not significantly improve implicit anti-fat bias. In another study that used a similar design, Poustchi et al. [37] showed the educational film followed by an interactive discussion among 64 second- and third-year medical students. Pre-post assessment showed improvement in medical student’s beliefs and stereotyping about obesity but no change in the perceptions and attitudes toward obese persons. These studies suggest that more intensive and targeted interventions are needed to address anti-obesity bias. Miller et al. [7] recommends a comprehensive change in curriculum that would include educating students about the impact of implicit bias on patient care, giving students multiple opportunities to reflect on their biases, and practicing strategies for minimizing the impact of those biases on their patient interactions and treatment decision.

Our study shows that confidence in communication skills and empathy toward patients with obesity can be improved with a brief encounter. National studies in the U.S. have shown that obesity counseling rates remain low among health care professionals [17,38]. A recent audio-recorded study among 39 urban primary care physicians also showed that physicians demonstrate less emotional rapport with overweight and obese patients compared to healthy weight patients [39]. Several other studies have demonstrated that physician’s acknowledgement of the patient’s weight [40] and physician diagnosis of overweight status [41] is associated with increased desire and attempts to lose weight. Currently, there is no clearly established method for telling patients they are overweight or obese [42]. However, initiating talk about weight is an interactive process, with information sharing between patient and physician. Recently, the American Medical Association issued a pamphlet titled *Weigh What Matters*, a family prevention program designed to provide resources needed to address weight with patients and their families [43]. The pamphlet contains several additional conversation starters to broach this sensitive topic.

There are limitations to the present study. One of the limitations is the use of a newly developed questionnaire. Items were selected from review of existing surveys from the medical literature that assessed provider attitudes and believes about obesity. However, reliability and validity of the items and derived scales will need further evaluation. Another limitation is the lack of a control group that would have strengthened the assertion that the SP intervention and associated learning activities led to the reported changes. The two follow up items on long-term effect rely on self-report and lack the methodological strength that the other items have by administering them at multiple time points. Sustainability of positive changes in confidence and attitudes regarding obese patients beyond two years is not certain. We also relied on self-reported attitudes and beliefs rather than observations. Although one of the stated outcomes of the study was to address anti-obesity bias, the small group faculty did not receive training in stigma or bias, and may have harbored bias themselves. We were also unable to differentiate which learning activity, i.e., pre-reading of two articles on obesity communication and anti-obesity bias, guided reflection and discussion in the small group, and the actual SP encounter, led to the immediate change in scale scores. The influence of other experiences over the subsequent year on obesity communication, empathy and bias were not measured. Finally, the study was conducted at one school and may not be generalizable. However, the incorporation of standardized patients into medical education is a well-established educational intervention.

In conclusion, this study has shown that medical student attitudes, beliefs and comfort in communication skills regarding obesity can be improved after a structured educational intervention that included a SP encounter. Immediate improvements were seen in three scales -- stereotyping, empathy and confidence, although one-year improvements were only seen for empathy and confidence. More intensive and targeted interventions are needed to address anti-obesity bias. Future studies will need to be conducted to evaluate the longer-term effect of these changes on the provision of obesity care.

## Competing interest

There were no competing financial interests related to this work.

## Authors’ contributions

RFK conceived the design of study, was involved in drafting and revising the manuscript, and gave final approval of the version to be published. DMZ participated in acquiring and analyzing the data, was involved in drafting and revising the manuscript, and gave final approval of the version to be published. JMF analyzed the data and performed scale analysis, was involved in drafting and revising the manuscript, and gave final approval of the version to be published. MY participated in training the standardized patients, acquiring survey data, was involved in drafting and revising the manuscript, and gave final approval of the version to be published.

## Pre-publication history

The pre-publication history for this paper can be accessed here:

http://www.biomedcentral.com/1472-6920/14/53/prepub
